# HS‐GC‐IMS detection of volatile organic compounds in Acacia honey powders under vacuum belt drying at different temperatures

**DOI:** 10.1002/fsn3.2364

**Published:** 2021-06-24

**Authors:** Duo Feng, Jing Wang, Yue He, Xiao‐jiao Ji, Hui Tang, Yong‐mei Dong, Wen‐jie Yan

**Affiliations:** ^1^ College of Biochemical Engineering Beijing Union University Beijing China; ^2^ Institute of Food and Nutrition Development Ministry of Agriculture and Rural Affairs Beijing China; ^3^ Beijing Tongrentang bee products (Jiangshan) Co., Ltd Jiangshan China

**Keywords:** Acacia honey powder, fingerprint similarity, HS‐GC‐IMS, PCA, vacuum belt drying

## Abstract

Honey is a commodity of great nutritional value, but deep‐processed honey products are uncommon. Herein, we used vacuum belt dryer to dry Acacia honey at 60°C, 70°C, and 80°C, prepared it into powder, and analyzed its volatile compound differences. We established HS‐GC‐IMS method to detect the volatile organic compounds (VOCs) of these three Acacia honey powders (AHPs). In total, 77 peaks were detected, and 23 volatile compounds were identified, including eight aldehydes, six ketones, three furans, one alcohol, one phenol, one lactone, one ester, one acid, and one nitrile. Moreover, principal component analysis (PCA) and fingerprint similarity analysis based on the Euclidean distance distinguished the three heating temperature treatments. Clearly, it was concluded that there are significant differences in volatile substances at different tested temperatures, and when the AHP was incubated at 80°C, more volatile compounds were detected.

## Graphical abstract

HS‐GC‐IMS detected volatile substances of honey powders. Honey powder with 80℃ contained more aldehydes, ketones, and furans.

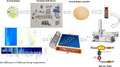

## 1 INTRODUCTION

Rich in nutrients and high medical value, honey is a natural sweet substance and is widely used in food processing, medical treatment, and brewing industries. However, the quality of honey is often affected by the seasonal temperature. The high temperature in the summer can lead to the production of acid by fermentation of the reducing sugars in the honey, and in winter, low temperature can cause incomplete crystallization, which affects its readjustment and quality uniformity (Ju & Miu, 2009). For such reasons, transforming liquid honey to solid form is a preferred option in various countries; for example, the honey powder can often be found in their market. Comparing to liquid form, honey powder is easy to store and transport, and has good antioxidant activity, low moisture content, and high solubility (Leyva‐Moguel et al., 2019).

The new low‐temperature continuous vacuum belt dryer adopts new nonstick belt materials, multistage temperature control, automatic vacuum adjustment, and a new structure. In the continuous drying system, products are heated by conduction or radiation in a vacuum state along a moving belt (Xu et al., 2013). Therefore, it has advantages including lower energy consumption, shorter drying time, lower cost, and minimal loss of volatile components. And the final product has lower moisture content, good flowability, higher hydrogen peroxide scavenging activity (Liu et al., 2011), and less thermal damage (Xu et al., 2016); therefore, it is optimized for the drying of solid and liquid materials. In modern food processing industries, vacuum belt drying is currently being used for low fat tortilla chips (Xu et al., 2013), muscadine pomace (Vashisth et al., 2011), apple pomace (Yan & Kerr, 2013), blueberry powder (Kim & Kerr, 2013), tomato powder (Xu et al., 2016), panax notoginseng (Liu et al., 2011), and red beetroot powders (Kerr & Varner, 2020). However, most of the ongoing researches are focusing on the physicochemical parameters, content of total phenol, and antioxidant activity of vacuum belt‐dried products, and it has not been used in the processing.

Headspace–gas chromatography–ion mobility spectroscopy (HS‐GC‐IMS) is fast (3–10 min), highly sensitive (detection limit as low as ppbv), highly selective, highly automated, of rapid response speed, and good stability (normal pressure work) equipment, without preparation and direct headspace sampling. It can conduct the qualitative analysis of a single compound and can also be used for rapid and result‐oriented analysis of the GC‐IMS 2D spectrum of the sample. Nowadays, the device has been used to distinguish the honey origins from different flowers by volatile substances (Schuhfried et al., 2016); or has been used for the classification and adulteration of different honeys (Schwolow et al., 2019; Wang, Yang, et al., 2019); and has been used to differentiate traditional honey and organic honey; it can be served as the supplement analysis tool for nuclear magnetic resonance (NMR) to distinguish honey from different flower sources (Gerhardt et al., 2018).

In this study, HS‐GC‐IMS was used to detect volatile organic compounds (VOCs) of honey powder with vacuum belt drying at different temperatures. In addition, PCA and fingerprint similarity based on the Euclidean distance were used to determine the consequences of temperature effects during honey powder processing. Finally, the knowledge obtained from this study has the potential to be utilized to improve the industrial process of honey powder production.

## 2 MATERIALS AND METHODS

### 2.1 Preparation of samples

Acacia honey was provided by Beijing Tongrentang Bee Products (Jiangshan) Co., Ltd, Jiangshan. Acacia honey was harvested in 2019. Generally, at room temperature, it has a thick fluid‐like shape; fresh Acacia honey is white in water and the color may be deepened when placed; and the content of glucose and fructose is more than 60 g/100 g, and sucrose is less than 5g/100 g. 5‐Hydroxymethylfurfural (HMF) is less than 10 mg/kg, and the HMF content was found to be within 4 h of heating at 80°C (Lu et al., 2006).

First, vacuum belt dryer (XMVBD 2‐4‐2; Changzhou Shinma Drying Technology Co., Ltd, Jiangsu, China) was vacuumed, and the Acacia honey sample was poured into the feeder. Then, the temperature of the vacuum drying chamber was set to 60°C, 70°C, and 80°C. After 70 min, the sample was cut into small pieces in the process of slow transmission. Finally, the samples were crushed into powder and vacuum packed for standby.

The abbreviation of Acacia honey powder under different processes are as follows: VBD‐AHP1 (after 60℃ vacuum belt drying of Acacia honey powder); VBD‐AHP2 (after 70°C vacuum belt drying of Acacia honey powder); and VBD‐AHP3 (after 80°C vacuum belt drying of Acacia honey powder).

### 2.2 HS‐GC‐IMS system

Headspace sampling conditions were set as follows: 2.0 g sample was placed in a 20 mL headspace bottle and incubated at 80°C for 20 min. The centrifuge speed was 500 rpm, and the temperature of inject needle was 85°C, and 500 μL sample was injected.

The GC conditions were set as follows: The gas chromatographic preseparation was performed on a FS‐SE‐54‐CB‐1 (15 m × 0.53 mm) capillary column at 60°C, the analysis time was 30 min, the carrier gas was N_2_ (purity ≥99.999%), and the flow rate was 0 ~ 2 min‐2 ml/min, 2 ~ 10 min‐2 ~ 10 ml/min, 10 ~ 20 min‐10 ~ 100 ml/min, and 20 ~ 30 min‐100 ~ 150 ml/min.

IMS conditions were set as follows: The temperature of the IMS ionization chamber was 45°C, the drift gas was N_2_ (purity ≥99.999%), and the flow rate was set to 150 ml/min.

### 2.3 Statistical analysis

Statistical data analysis was performed by Laboratory Analytical Viewer (LAV) and GC‐IMS Library Search software from different angles.

## 3 RESULTS

### 3.1 HS‐GC‐IMS plots of different treatments of honey powders

**FIGURE 1:**
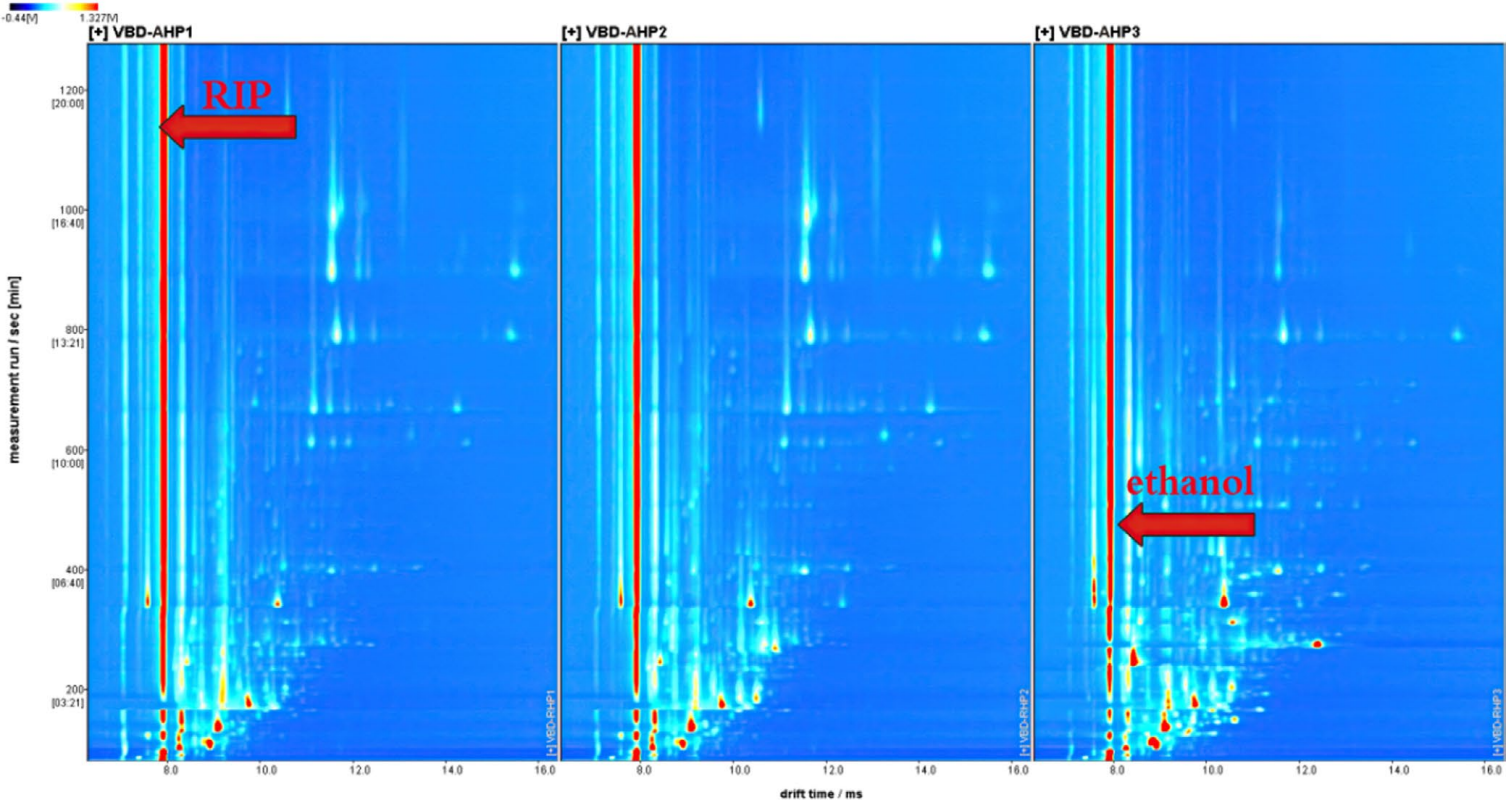
HS‐GC‐IMS plot of Acacia honey powders

In this study, HS‐GC‐IMS was used to analyze the VOCs of Acacia honey powders under different temperatures during vacuum belt drying processing. From Figure 1, the ordinate represents the retention time of the gas chromatography, and the abscissa represents drift time. When the drift time is between 7.92 and 7.93 ms, there is a reaction ion peak (RIP). Moreover, when the drift time is between 8.3 and 8.8 ms, there is an ethanol peak marked separately, with a higher signal response intensity.

**FIGURE 2:**
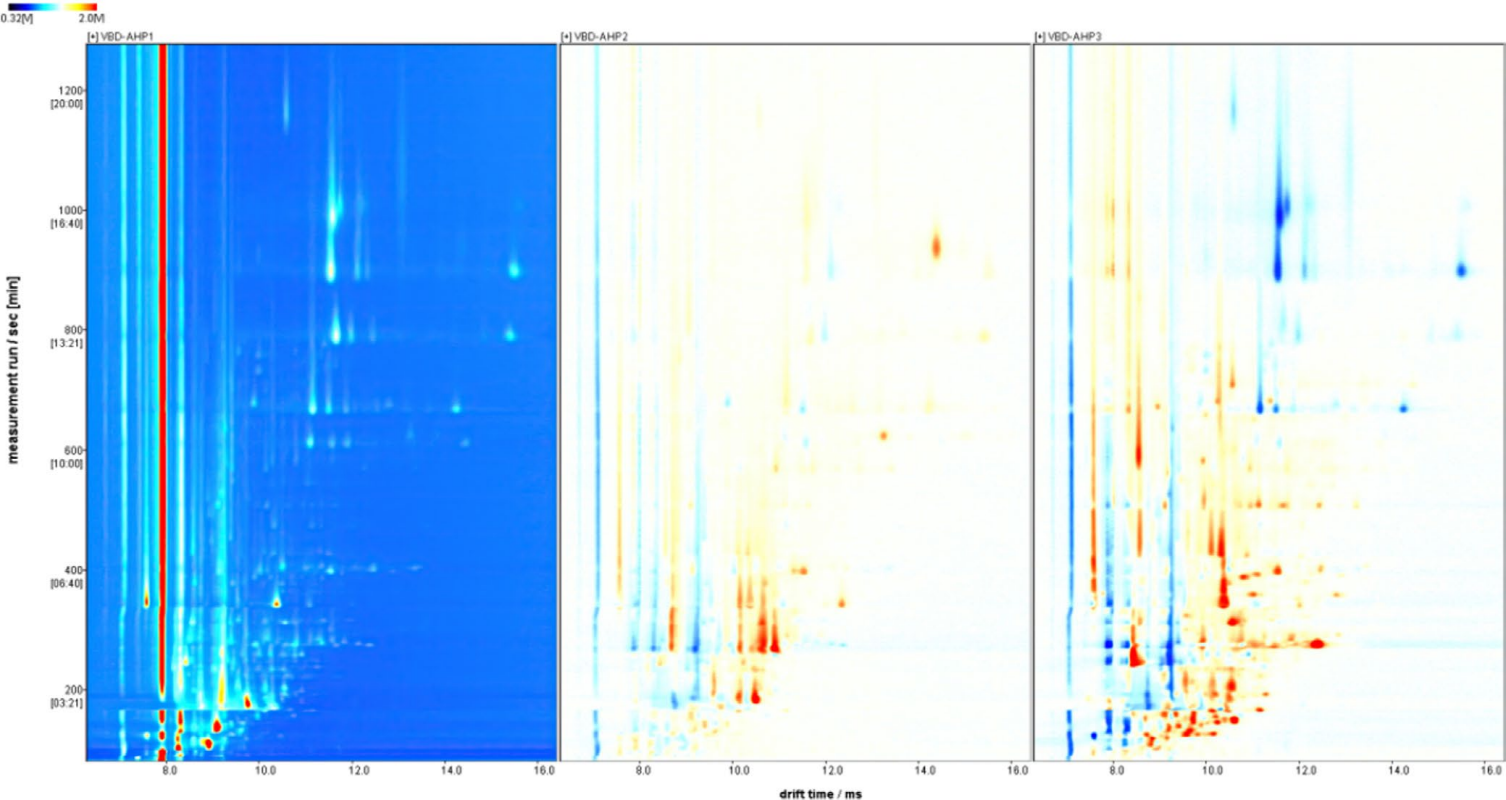
HS‐GC‐IMS plot in difference comparison mode of Acacia honey powders

In order to compare the differences between this three samples in more detail, the different comparison modes (Figure 2): Select the spectrum of VBD‐AHP1 as the reference, and subtract the reference from the spectra of the two other samples. If the two VOCs are the same, the deducted background is white. And red means the concentration of the substance is higher than the reference, while blue means lower. The brighter the color, the higher the content, and vice versa. It can be observed from Figure 2 that there was little difference between VBD‐AHP1 and VBD‐AHP2. When the drift time is between 8.0 and 9.5, the content of VOCs in VBD‐AHP2 was lower than VBD‐AHP1, and later, when the drift time is 10.0, VBD‐AHP2 was higher, but the difference was not significant. For VBD‐AHP3, the contents of volatile substances were more complicated than the former two, so further judgment was needed. However, more volatile compounds were found in VBD‐AHP3, maybe that high temperature promoted some fewer volatile compounds (Plutowska et al., 2011).

### 3.2 Identification of VOCs from different treatments of Acacia honey powders

**FIGURE 3:**
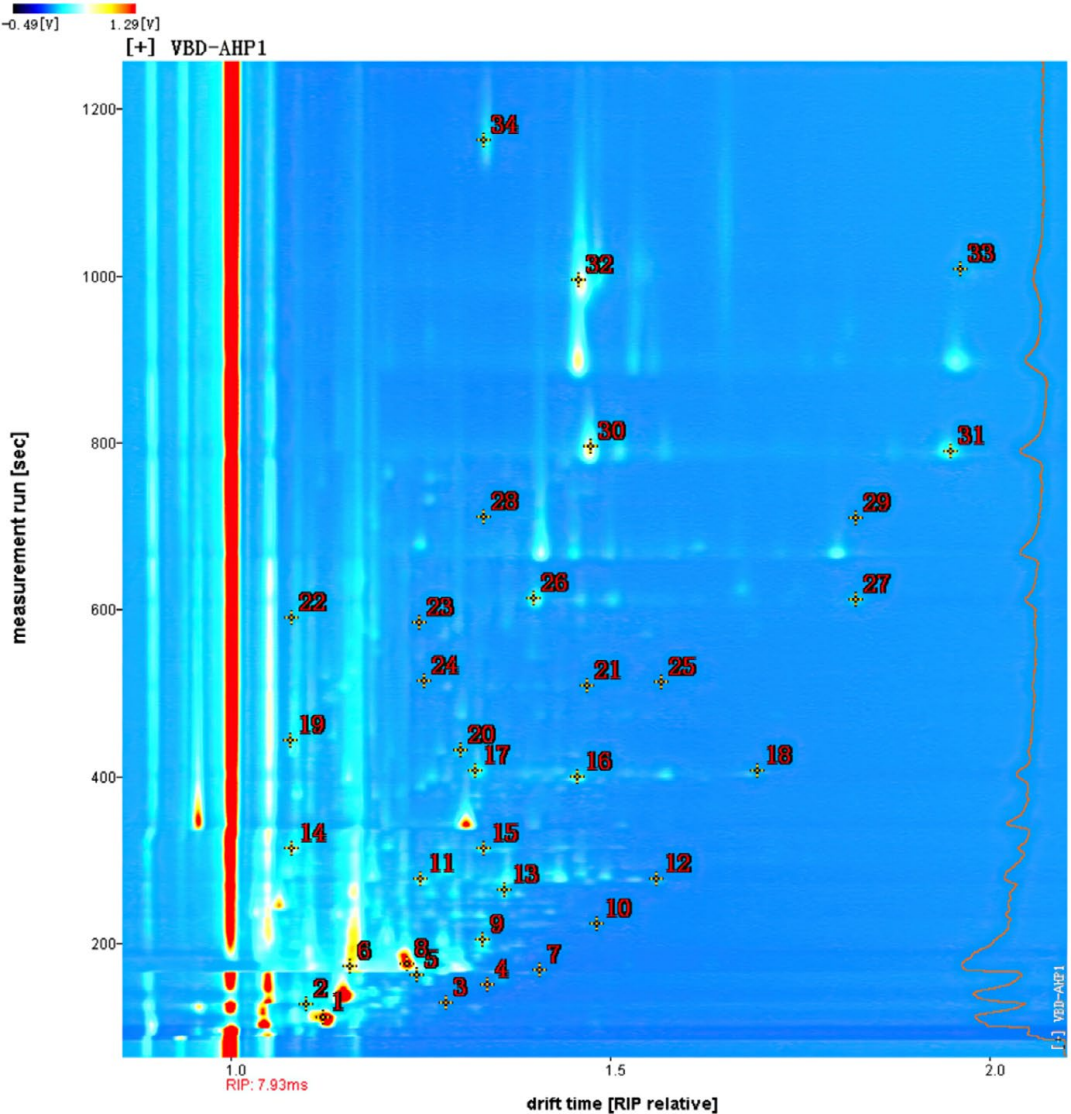
HS‐GC‐IMS identification plot of Acacia honey powders

The aroma components of Acacia honey contain alcohols (14.41 ng/ml), alkanes (4.34 ng/ml), esters (3.31 ng/ml), acids (3.06 ng/ml), aldehydes (2.45 ng/ml), furan, benzene and its derivatives (0.93 ng/ml), and ketones (0.70 ng/ml) (Pei et al., 2014). In this research, HS‐GC‐IMS was used to detect the VOCs of Acacia honey powder with vacuum belt drying at different temperatures. The qualitative analysis of volatile components in Acacia honey powder is shown in Figure 3, in which the abscissa represents the drift time (normalized) and the ordinate represents the retention time, and the numbers correspond to the compounds in Table 1. A total of 77 peaks were detected and 23 volatile compounds were identified, including eight aldehydes, six ketones, three furans, one alcohol, one phenol, one lactone, one ester, one acid, and one nitrile. Table 1 lists the qualitative results, including the compound name, CAS number, molecular weight (MW), the Retention Index (RI), the retention time (RT), and the drift time (DT).

**TABLE 1:** Qualitative results of VBD‐AHP with different temperatures

Count	Compound	CAS#	Formula	MW	RI	Rt [sec]	Dt [RIPrel]	Comment
1	Acetone	C67641	C3H6O	58.1	521.1	111.572	1.1223	
2	Methylpropanal	C78842	C4H8O	72.1	556.9	127.652	1.10043	Monomer
3	Methylpropanal	C78842	C4H8O	72.1	558.4	128.295	1.28429	Dimer
4	Ethyl acetate	C141786	C4H8O2	88.1	607.1	150.165	1.33937	
5	3‐Butenenitrile	C109751	C4H5N	67.1	633.8	162.171	1.24622	
6	2‐Methylbutanal	C96173	C5H10O	86.1	656.8	172.462	1.15794	Monomer
7	2‐Methylbutanal	C96173	C5H10O	86.1	645.8	167.531	1.40822	Dimer
8	Hydroxyacetone	C116096	C3H6O2	74.1	662	174.821	1.23408	
9	Acetoin	C513860	C4H8O2	88.1	709.4	204.608	1.33238	
10	3‐Methylbutanol	C123513	C5H12O	88.1	731	223.144	1.48396	
11	Hexanal	C66251	C6H12O	100.2	791.5	277.716	1.25066	Monomer
12	Hexanal	C66251	C6H12O	100.2	791.2	277.347	1.5633	Dimer
13	2,3‐Butanediol	C513859	C4H10O2	90.1	778.1	263.579	1.36237	
14	Furfurol	C98011	C5H4O2	96.1	822	314.521	1.08161	Monomer
15	Furfurol	C98011	C5H4O2	96.1	821	313.226	1.33504	Dimer
16	Cyclohexanone	C108941	C6H10O	98.1	892.1	399.138	1.45802	
17	Heptanal	C111717	C7H14O	114.2	896.6	407.34	1.32386	Monomer
18	Heptanal	C111717	C7H14O	114.2	896.5	407.161	1.69613	Dimer
19	Butyrolactone	C96480	C4H6O2	86.1	916.5	443.719	1.08012	Monomer
20	Butyrolactone	C96480	C4H6O2	86.1	909.7	431.227	1.30466	Dimer
21	Benzaldehyde	C100527	C7H6O	106.1	952	508.537	1.47138	
22	Phenol	C108952	C6H6O	94.1	996.7	590.146	1.08129	
23	2‐Pentylfuran	C3777693	C9H14O	138.2	993.3	584.028	1.24988	
24	5‐Methyl−2‐furanmethanol	C3857258	C6H8O2	112.1	955.8	515.423	1.25676	Monomer
25	5‐Methyl−2‐furanmethanol	C3857258	C6H8O2	112.1	954.4	512.838	1.56863	Dimer
26	Octanal	C124130	C8H16O	128.2	1,008.6	613.633	1.4	Monomer
27	Octanal	C124130	C8H16O	128.2	1,008	612.341	1.82611	Dimer
28	(E)−2‐Octenal	C2548870	C8H14O	126.2	1,058.8	711.843	1.33473	Monomer
29	(E)−2‐Octenal	C2548870	C8H14O	126.2	1,057.8	709.905	1.82611	Dimer
30	*n*‐Nonanal	C124196	C9H18O	142.2	1,101.4	795.193	1.47616	Monomer
31	*n*‐Nonanal	C124196	C9H18O	142.2	1,099.1	790.67	1.95123	Dimer
32	2‐Decanone	C693549	C10H20O	156.3	1,203.8	995.554	1.46005	Monomer
33	2‐Decanone	C693549	C10H20O	156.3	1,210.5	1,008.828	1.96436	Dimer
34	Phenylacetic acid	C103822	C8H8O2	136.1	1,289.4	1,163.198	1.3341	

Heat treatment can increase aldehydes and ketones (Li et al., 2012). And aldehydes were derived from the auto‐oxidation of lipid, while ketones were mainly derived from the thermal oxidation or degradation of unsaturated fatty acids (Liu et al., 2020). Surprisingly, n‐Nonanal was only found in Acacia honey, and also contained its homologues heptanal and octanal, which had sweet citrus flavor (Plutowska et al., 2011). Moreover, Escriche et al. (2009) found that heat treatment significantly changed 29 compounds, 20 of which belonged to the alcohol and aldehyde family. Among them, 2,3‐butanediol was detected and its content was increased by pasteurization. 2,3‐Butanediol can be prepared from sugar, molasses, malt syrup, or alcohol mother liquor as raw materials through biological fermentation. Manuel Viuda‐Martos et al. (2010) detected ten honey samples and found that honey was characterized by high level of benzene and furan‐related compounds. Escriche et al. (2009) and Castro‐Va´zquez et al. (2007) suggested treating benzene and phenolic compounds as characteristic compounds of honeydew honeys. Of note was that, in this study, a large number of benzene compounds (benzaldehyde, phenol, phenylacetic acid) were also found. In addition, Acacia honey contained a lot of esters, accounting for 32.43% of aroma components (Pei et al., 2014). But a small amount of ethyl acetate was detected in Acacia honey (Wang et al., 2021), may be derived from the interaction of alcohol and free fatty acids produced by lipid oxidation in the sample (Zhang et al., 2019). Acacia honey also contained phenylacetaldehyde (1.96 ng/ml) (Pei et al., 2014), which was heated and oxidized to generate phenylacetic acid. The detected 3‐butenenitrile belonged to the category of nitriles, which may be caused by prolonged contact between packaging materials and honey powders.

### 3.3 Gallery plot of different treatments of Acacia honey powders

**FIGURE 4:**
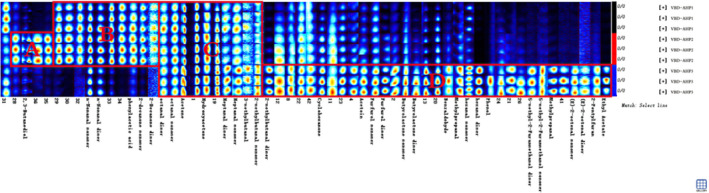
Fingerprint of volatile compounds of Acacia honey powders

In order to clearly compare the specific volatile substance differences in each group of VBD‐AHP samples, all peaks are selected below for fingerprint comparison. Each row in the figure represents all the signal peaks selected in the sample, and each column represents the signal peak of the same VOC in different samples. The complete VOC information of each sample and the difference in VOCs between samples can be seen from the fingerprint (Figure 4). The individual dot represents a volatile substance, and the color extent represents the content levels of the volatile substance, the brighter the color, the higher the content. In the fingerprint plot, unidentified substances are represented by numbers, and some substances with monomer and dimer morphology were detected. Methylpropanal, 2‐methylbutanal, hexanal, furfurol, heptanal, butyrolactone, 5‐methyl‐2‐furanmethanol, octanal, (E)‐2‐octenal, n‐Nonanal, and 2‐decanone had the forms of monomer and dimer.

It can be seen from Figure 4 that more types of volatile compounds were detected in VBD‐AHP3. A was the characteristic region of VBD‐AHP2, including 2,3‐butanediol; N‐Nonanal, phenylacetic acid, and 2‐decanone were the characteristic volatile substances and marked as B area in VBD‐AHP1 and VBD‐AHP2; the VOCs of the C area were detected in all treatments, including octanal, acetone, hydroxyacetone, heptanal, and 3‐methylbutanal; and D represented the VBD‐AHP3 characteristic area. Studies have found that the increase in temperature was conducive to the formation of aldehydes and ketones (Li et al., 2012), while not conductive to the formation of acids (Liu et al., 2019). Therefore, the content of phenylacetic acid in VBD‐AHP3 was less than the first two. Ethyl acetate was the main component of the head fraction fermentation of honey spirits, and maybe the temperature of VBD‐AHP3 was close to its presteaming temperature to produce a small amount of ethyl acetate (Anjos et al., 2017). Meanwhile, only benzaldehyde increased at 80 ℃ (Escriche et al., 2009), and heat treatment could increase the contents of acetone and furfurol. Furthermore, furan compounds were only detected in VBD‐AHP3, which may due to the increase in sugar degradation and Maillard reaction rate. And furan derivatives can be used as a good indicator for heat treatment and storage of honey (Escriche et al., 2009).

### 3.4 Cluster analysis of Acacia honey powders

Principal component analysis (PCA) is a multivariate statistical method used to examine the correlation between multiple variables, constitutes a powerful visualization tool, provides a method to reduce the dimensionality of the data, and can eliminate unnecessary information (Chen et al., 2020). In order to analyze the problem comprehensively, PCA is applied to these variables. Generally, when the cumulative contribution rate of PC1 and PC2 reaches 60%, PCA model is considered as the preferred separation model (Wu et al., 2015). PCA had been used to distinguish honey from different floral origins, different varieties of honey, and discrimination between conventional honey and organic honey (Schuhfried et al., 2016; Schwolow et al., 2019; Wang, Yang, et al., 2019). However, less research for the processes of honey powders, especially on the flavor of them under vacuum belt drying with different temperatures.

**FIGURE 5:**
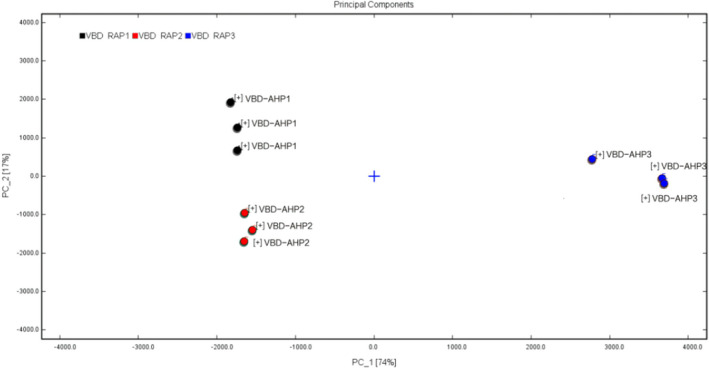
PCA of VBD‐AHPs

In this study, PCA was used to distinguish VBD‐AHP at different temperatures. A total cumulative contribution was 91%, of which PC1 was 74% and PC2 was 17%. In addition, it can be observed from Figure 5 that VBD‐AHP1 can be well‐distinguished by negative score values of PC1 and positive scores of PC2. And VBD‐AHP2 can be confirmed by negative scores of PC1 and PC2. Meanwhile, VBD‐AHP3 can be separated by positive scores of PC1. It should be noted that the distance of VBD‐AHP1 and VBD‐AHP2 was relatively close, which can confirm that there was no significant difference between them. However, both of them were far away from VBD‐AHP3, which can prove that the components were obviously different.

**FIGURE 6:**
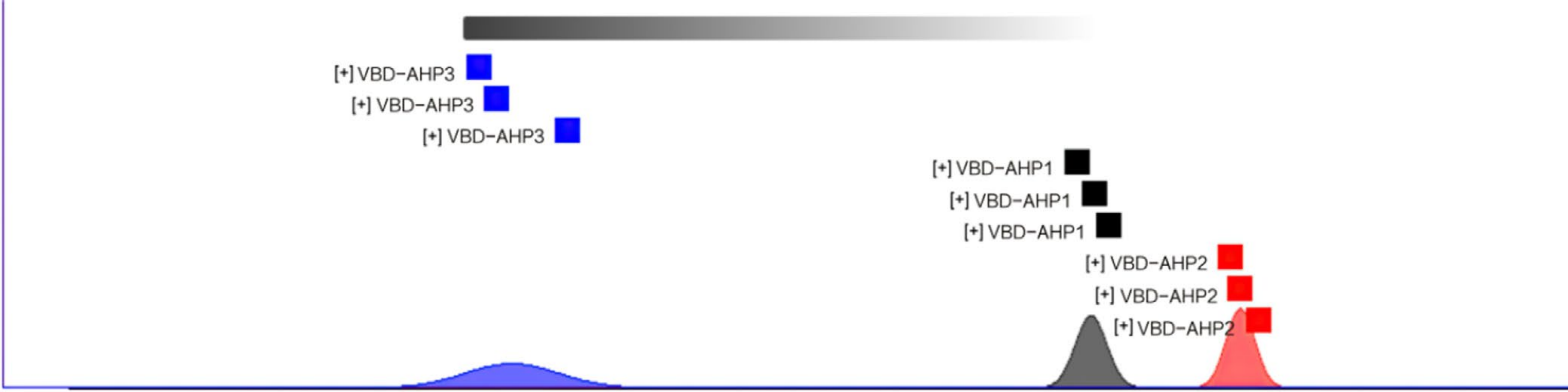
Fingerprint similarity based on the Euclidean distance of VBD‐AHPs

Meanwhile, the analysis of fingerprint similarity based on the Euclidean distance judged the difference in the samples. This method is a cluster analysis method based on distance discrimination, which refers to the true distance between two points in space, or the natural length of the vector (i.e., the distance from the point to the origin) (Tang, 2019), reflects the degree of intimacy between the research subjects (Li et al., 2008). Wang, Zhou, et al. (2019) used the square Euclidean distance measurement method to cluster analysis on the gas‐phase matching data of 38 honey samples from 4 different nectar sources, and found that these honeys could be clustered into one category, respectively. Figure 6 shows the fingerprint similarity based on Euclidean distance, and Table 2 shows the values of Euclidean distance between the three.

**TABLE 2:** Euclidean distance of VBD‐AHPs

Full distance	[+] VBD‐AHP1	[+] VBD‐AHP1	[+] VBD‐AHP1	[+] VBD‐AHP2	[+] VBD‐AHP2	[+] VBD‐AHP2	[+] VBD‐AHP3	[+] VBD‐AHP3	[+] VBD‐AHP3
[+] VBD‐AHP1	0	292,205.5899	1,040,817.066	2,273,235.224	3,852,584.406	3,063,766.191	10,362,993.91	14,899,633.93	14,534,843.23
[+] VBD‐AHP1	292,205.5899	0	341,636.7061	1,728,892.43	3,004,368.358	2,298,163.793	9,586,089.602	13,715,247.37	13,377,256.32
[+] VBD‐AHP1	1,040,817.066	341,636.7061	0	1,689,302.885	2,707,816.728	1,947,336.629	9,486,166.598	13,401,443.45	13,082,500.49
[+] VBD‐AHP2	2,273,235.224	1,728,892.43	1,689,302.885	0	646,520.2193	194,545.7289	9,545,363.99	12,998,646.87	12,543,511.08
[+] VBD‐AHP2	3,852,584.406	3,004,368.358	2,707,816.728	646,520.2193	0	376,649.7102	10,295,983.65	13,284,950.17	12,959,422.54
[+] VBD‐AHP2	3,063,766.191	2,298,163.793	1,947,336.629	194,545.7289	376,649.7102	0	9,521,521.662	12,645,846.39	12,154,276.39
[+] VBD‐AHP3	10,362,993.91	9,586,089.602	9,486,166.598	9,545,363.99	10,295,983.65	9,521,521.662	0	1,380,639.112	1,327,675.052
[+] VBD‐AHP3	14,899,633.93	13,715,247.37	13,401,443.45	12,998,646.87	13,284,950.17	12,645,846.39	1,380,639.112	0	350,826.5962
[+] VBD‐AHP3	14,534,843.23	13,377,256.32	13,082,500.49	12,543,511.08	12,959,422.54	12,154,276.39	1,327,675.052	350,826.5962	0

We can find that VBD‐AHP1 and VBD‐AHP2 were relatively close, and the average Euclidean distance between them was 2,507,274.071. Meanwhile, the average Euclidean distance between VBD‐AHP1 and VBD‐AHP3 was 12,494,019.43 and VBD‐AHP2 and VBD‐AHP3 was 11,772,169.19. So, the difference in VBD‐AHP3 was more significant than the first two.

## 4 DISCUSSION AND CONCLUSION

Generally, liquid Acacia honey has a thick fluid‐like shape; fresh Acacia honey is colourless and the color may be deepened when it placed; moreover, it has light fragrance of sophora flower and is not easy to crystallize, and no impurities were visible with normal vision. Meanwhile, the content of glucose and fructose is more than 60 g/100 g, sucrose is less than 5 g/100 g, and maltose is less than 3 g/100 g. From the visual analysis, brownish‐yellow amorphous powder of AHP at 60, 70°C, while light yellow amorphous powder of AHP at 80°C. And AHP3 is finer and less dense than those at 60°C and 70°C. Most importantly, honey powder would retain pure honey flavor and is a natural sweetener, which is more convenient to carry than liquid honey, avoiding the waste of liquid honey and environmental hygiene problems, reducing storage space, and prolonging preservation time. Therefore, the study of AHP is crucial and essential.

Because honey powder is a deep processing product of honey, and nowadays, the processing of honey powder is monotonous, mainly freeze‐dried and roller cylinder‐dried. So, we considered whether vacuum belt drying can be applied in the honey industry, and we looked up the literature and found that, for materials with high stickiness, easy agglomeration, thermoplastic, and thermosensitive properties, the best option was vacuum‐belt dryer. Therefore, in order to analyze its process parameters, we considered three different temperatures of vacuum belt drying, mainly to detect the differences in VOCs between the three honey powders, and to identify differences in flavor aspects that consumers pay attention to.

The HMF in Acacia honey is mainly produced by amino acids and glucose or fructose in honey undergoing the Maillard reaction under acidic conditions (Kowalski et al., 2013). Since HMF can cause irritation to human eyes, mucous membranes, skin, etc., and can cause cell and gene mutations, excessive intake can result in poisoning and even initiate cancer (Ma et al., 2019). Therefore, HMF in international trade of honey belongs to the mandatory detection indicator, which states that its content should be ≤40 mg/kg. Our raw material acceptance criteria indicate that the HMF is less than 10 mg/kg, and the HMF content was found to be within 4 h of heating at 80°C (Lu et al., 2006). In addition, Lu et al. (2006) found that the formation rate of HMF was buckwheat honey >jujube honey >acacia honey >locust honey, and proposed that during honey thermal processing treatment, heating temperature should be controlled within 80°C to prevent excessive HMF content. Furthermore, Wang et al. (2018) surveyed to obtain commercially available Acacia honey in Chengdu, Sichuan Province, China, the number of detected was 22, the eligibility rate was 100%, and the mean content of HMF was 5.96 ± 2.93 mg/kg, 81.82% were in the range of 0–10 mg/kg, and 18.18% were in the range of 10.1–20 mg/kg.

In this study, HS‐GC‐IMS was used to detect VOCs of VBD‐AHP. A total of 77 peaks were detected and 23 volatile compounds were identified, including eight aldehydes, six ketones, three furans, one alcohol, one phenol, one lactone, one ester, one acid, and one nitrile. Since GC‐IMS is not able to detect alkane compounds, 43 signal peaks were not characterized in the current study, and these unknown peaks will be worthy to continue to be identified using GC‐MS or other detection methods in the future. The results showed that VBD‐AHP3 samples contained more aldehydes, ketones, and furans. In addition, PCA and fingerprint similarity based on the Euclidean distance can readily distinguish VBD‐AHP1, VBD‐AHP2, and VBD‐AHP3. The follow‐up improvements should focus on quantitative research of detecting volatile substances, so as to know the aroma threshold of the substance in honey powder, and providing theoretical support for the processing of honey powder with different flavors and, finally, for the industrial development of vacuum belt dryers used in honey powders.

## CONFLICT OF INTEREST

The authors declare no conflicts of interests.

## AUTHORS CONTRIBUTIONS

Duo Feng investigated the study, wrote the original draft, and involved in plot analysis. Jing Wang involved in formal analysis. Yue He investigated the study. Xiao‐jiao Ji validated the study. Hui Tang provided resources. Yong‐mei Dong visualized the data. Wen‐jie Yan wrote, reviewed, and edited the manuscript, supervised the data, acquired funding.
